# Supplementary material for: Robust charge-density-wave correlations in the electron-doped single-band Hubbard model
Source: Nat Commun. 2023 May 20;14:2889. doi: 10.1038/s41467-023-38566-7 (PMC10199951; doi:10.1038/s41467-023-38566-7)
Supplement: Supplementary file 1 — Supplementary Information [file 41467_2023_38566_MOESM1_ESM.pdf]

Supplementary Information for "Robust charge-density wave correlations in the electron-doped single-band Hubbard model"

Peizhi Mai,[1,2] Nathan S. Nichols,[3] Seher Karakuzu,[4,5] Feng Bao,[6]
Adrian Del Maestro,[7,8,9] Thomas A. Maier,[4] and Steven Johnston[7,8]

[1] *Computational Sciences and Engineering Division, Oak Ridge National Laboratory, Oak Ridge, Tennessee 37831-6494, USA*
[2] *Department of Physics and Institute of Condensed Matter Theory, University of Illinois at Urbana-Champaign, Urbana, Illinois 61801, USA*
[3] *Data Science and Learning Division, Argonne National Laboratory, Argonne, Illinois 60439, USA*
[4] *Computational Sciences and Engineering Division, Oak Ridge National Laboratory, Oak Ridge, Tennessee, 37831-6494, USA*
[5] *Center for Computational Quantum Physics, Flatiron Institute, 162 5th Avenue, New York, New York 10010, USA*
[6] *Department of Mathematics, Florida State University, Tallahassee, Florida 32306, USA*
[7] *Department of Physics and Astronomy, The University of Tennessee, Knoxville, Tennessee 37996, USA*
[8] *Institute of Advanced Materials and Manufacturing, The University of Tennessee, Knoxville, Tennessee 37996, USA*
[9] *Min H. Kao Department of Electrical Engineering and Computer Science, University of Tennessee, Knoxville, Tennessee 37996, USA*

(Dated: April 18, 2023)

Supplementary Note 1. ERROR ANALYSIS FOR THE CHARGE AND SPIN CORRELATIONS IN MOMENTUM SPACE

In this section, we show the DCA momentum-dependent static charge and spin correlations at the lowest temperature with error bar in Fig. S1. The double-peak features in the charge correlations of both hole (h)- and electron (e)-doped cases are clearly outside the error bar.

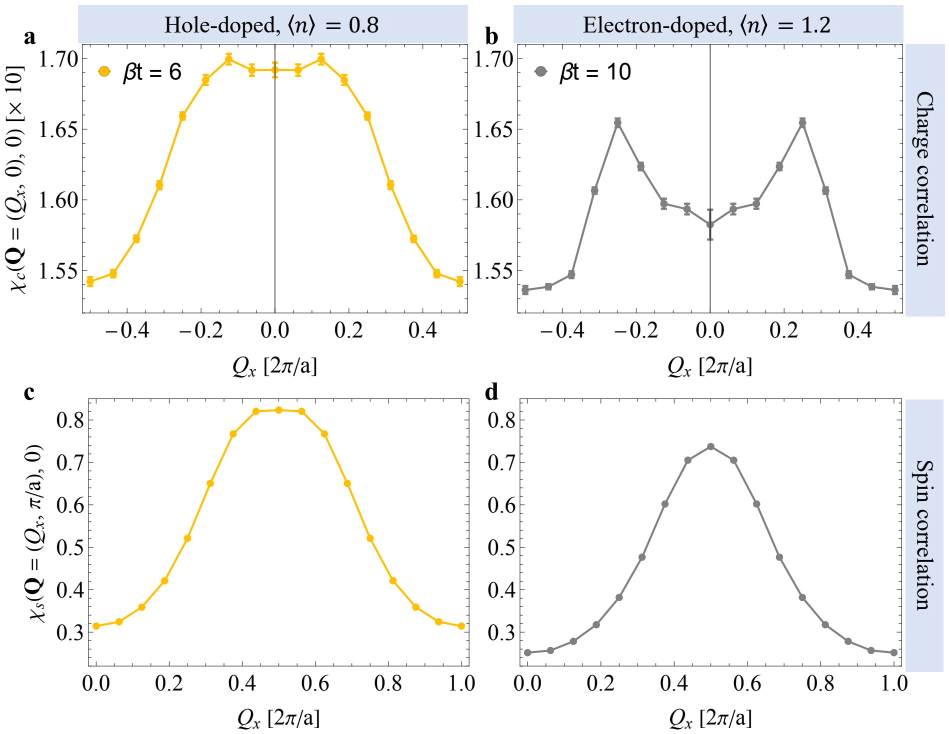

FIG. S1: **Static charge and spin correlations in momentum-space from DCA simulations.** Panels **a** and **b** show the static charge susceptibility $\chi_c(\mathbf{Q}, 0)$ along the $(Q_x, 0)$ direction, for h- and e-doped systems respectively at the their lowest temperatures. Panel **c** and **d** show the corresponding static spin susceptibility $\chi_s(\mathbf{Q}, 0)$ along the $\mathbf{Q} = (Q_x, 0.5)$ direction. These results correspond the real-space data in Fig. 2 of the main text with error bar.

Supplementary Note 2. DETERMINANT QUANTUM MONTE-CARLO SIMULATION FOR ELECTRON-DOPED SINGLE-BAND HUBBARD MODEL

We have performed determinantal quantum Monte-Carlo (DQMC) simulations for the charge correlations in the e-doped single-band Hubbard model on 16×4 clusters with periodic boundary conditions and $U/t = 6$. These calculations complement our DCA simulations and provide a cross-check on the nature of the charge correlations discussed in the main text. The results for the charge correlations are presented in Fig. S2(a,b) at $t' = -0.2, -0.3$, respectively, both at an inverse temperature $\beta = 4.5/t$. (DQMC is generally restricted to higher temperatures due to the more severe Fermion sign problem in in comparison to DCA.) Although the temperature is higher than that in the DCA results, we can already observe the formation of a checkerboard pattern in the middle of both panels (Fig. S2) and signals of unidirectional CDW components in Fig. S2(b). For comparison, we also show the DCA results with the same parameters except at lower temperature ($\beta = 8/t$), corresponding to the data in Fig. 3b of the main text. The DQMC and DCA results are consistent (particularly for $t' = -0.3$), with both methods finding a coexistence of a middle checkerboard and unidirectional CDW correlations.

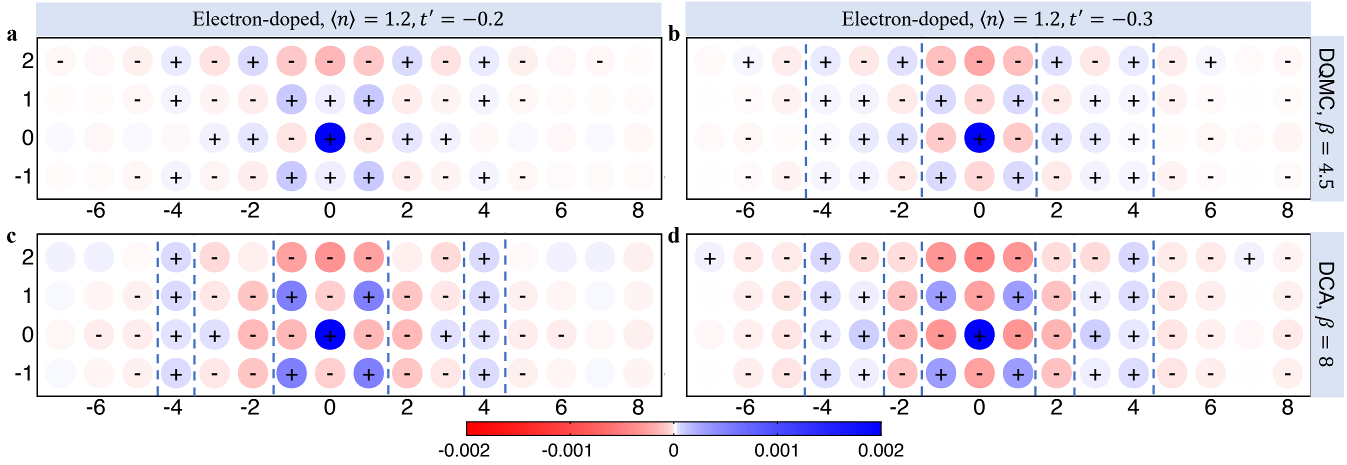

FIG. S2: **Static charge correlations in real-space from DQMC and DCA simulations.** Panels **a** and **b** plot $\chi_c(\mathbf{r}, 0)$ from DQMC simulations for the e-doped system ($\langle n \rangle = 1.2$) at $t'/t = -0.2$ and -0.3, respectively, both with $\beta = 4.5/t$. Panels **c** and **d** show the DCA charge correlation at $t'/t = -0.2$ and -0.3, respectively, both with $\beta = 8/t$. The interaction strength is $U/t = 6$ for all cases. $+$ and $-$ signs indicate the sign of the correlations whose absolute mean is larger than two standard errors. The dashed lines indicate the approximate nodes in charge stripe modulations.

Supplementary Note 3. ANALYTIC CONTINUATION

We used three independent methods to perform the analytic continuation of the dynamical spin [$S(\mathbf{Q},\omega)$] and charge [$N(\mathbf{Q},\omega)$] structure factors. Specifically, we used a parameter-free differential evolution algorithm [1], the method of Maximum Entropy [2], and stochastic optimization [3]. Fig. S3 compares the results obtained from these three methods. Here, $S(\mathbf{Q},\omega)$ is shown in the first column, $N(\mathbf{Q},\omega)$ is shown in the second column, and the sum of columns one and two is shown in the third column for reference. Results obtained using the evolution algorithm are shown in Figs. S3(a-c). Similarly, results for the maximum entropy method are shown in Figs. S3(d-f), and results for the stochastic optimization method are shown in Figs. S3(g-i). Note that the first row is identical to the results shown in Fig. 4 of the main text.

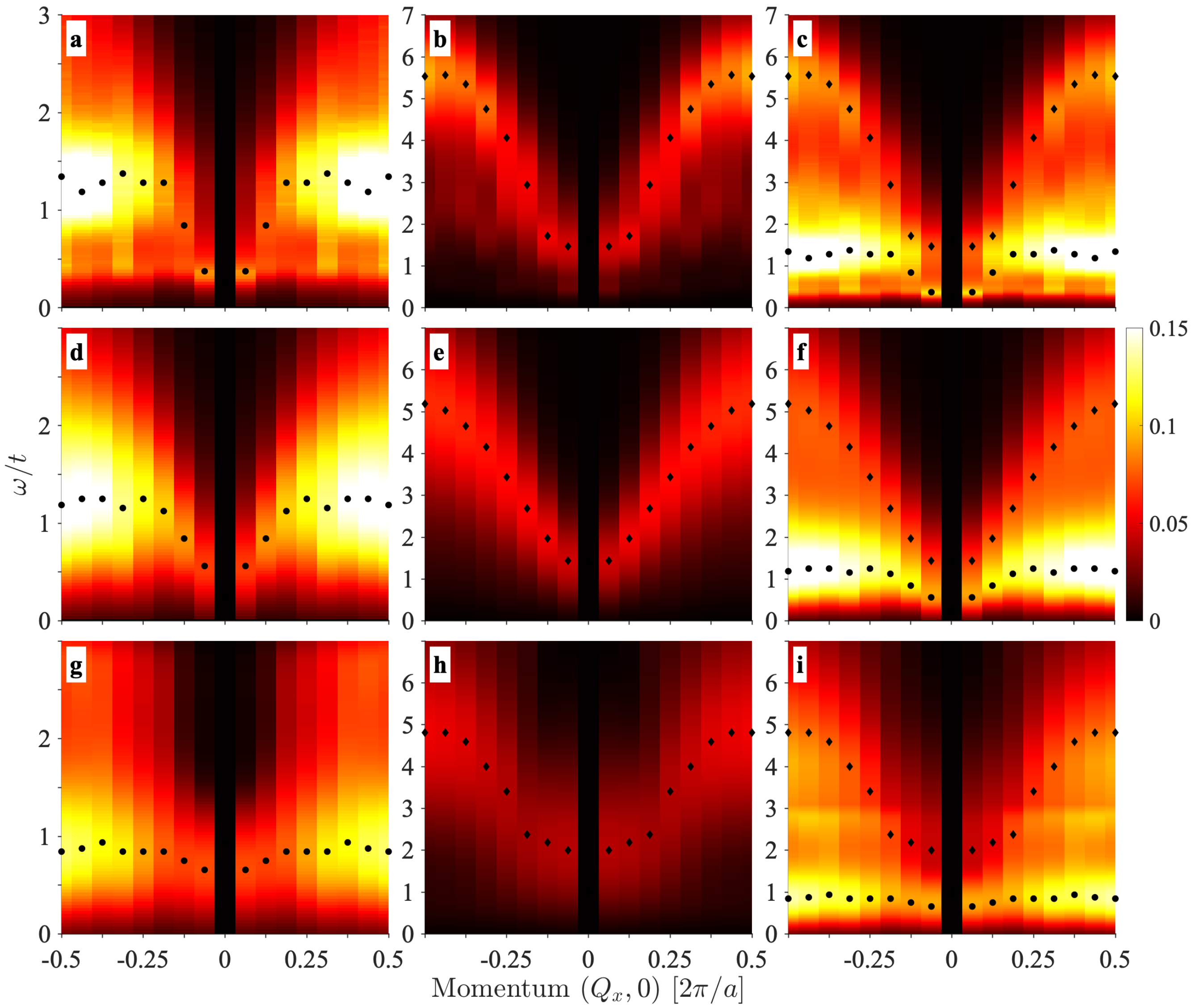

FIG. S3: A comparison of the dynamical spin $S(\mathbf{Q},\omega)$ (left column) and charge $N(\mathbf{Q},\omega)$ structure factors (middle column) and their sum $S(\mathbf{Q},\omega)+N(\mathbf{Q},\omega)$ (right column), obtained through analytic continuation using a differential evolution algorithm (top row), Maximum Entropy (middle row) and stochastic optimization (bottom row). All panels are plotted on the same color scale, as indicated on the right.

Supplementary Note 4. COMPARISONS OF THE FULL STATIC CHARGE SUSCEPTIBILITY TO WEAK COUPLING PICTURES

In this section, we compare the interacting static charge susceptibility for the e-doped model (see Figs. 1 and 3 of the main text) to the static charge susceptibility computed using the bare and interacting Green's functions (without and with random phase approximation (RPA)).

In the non-interacting limit, the charge susceptibility is given by the Lindhard formula

$$\chi_{\mathrm{c},G_0G_0}(\mathbf{Q},0) = \frac{1}{N_{\mathrm{sites}}}\sum_{\mathbf{k}} \frac{f(\varepsilon_{\mathbf{k}}) - f(\varepsilon_{\mathbf{k}+\mathbf{Q}})}{\varepsilon_{\mathbf{k}+\mathbf{Q}} - \varepsilon_{\mathbf{k}} + i\delta}, \tag{S1}$$

where $f(x) = 1/[e^{\beta(x-\mu)} + 1]$ is the Fermi-Dirac distribution function with the appropriate chemical potential μ for the targeted density $\langle n \rangle$. The calculation for Eq. (S1) is performed on an $N_{\mathrm{sites}} = 160 \times 40$ cluster to eliminate finite-size effects. In the interacting case, the charge susceptibility in Eq. (4) of the main text can be calculated using the single-particle Green's function within the bubble approximation and discarding the vertex corrections

$$\chi_{\mathrm{c},GG}(\mathbf{Q},0) = \frac{1}{\beta N_{\mathrm{sites}}}\sum_{\omega_n,\mathbf{k}} G(\mathbf{k}, i\omega_n) G(\mathbf{k}+\mathbf{Q}, i\omega_n). \tag{S2}$$

To compare the Lindhard result better and reduce the finite-size effects, we calculated $\chi_{\mathrm{c},GG}$ using the lattice Green function by interpolating the DCA cluster self-energy onto a 160×40 lattice [4]. With $\chi_{\mathrm{c},GG}$, we calculate the corresponding RPA susceptibility

$$\chi_{\mathrm{c,RPA}}(\mathbf{Q},0) = \frac{\chi_{\mathrm{c},GG}(\mathbf{Q},0)}{1 + U_{\mathrm{eff}}\, \chi_{\mathrm{c},GG}(\mathbf{Q},0)} \tag{S3}$$

with an effective coupling strength U_{eff} that is reduced from the bare U due to screening effects [5, 6].

The results of $\chi_{\mathrm{c},GG}$, χ_{c,G_0G_0} and $\chi_{\mathrm{c,RPA}}$ in the static limit ($\omega = 0$) are compared to the full static charge susceptibility χ_{c} obtained from DCA in Figs. S4-S6. Specifically, we show results for varying temperature (Fig. S4), density (Fig. S5), and next-nearest-neighbor hopping (Fig. S6) along the $(Q_x, 0)$ and $(Q_x, 0.5)$ directions, respectively.

Our first observation in Figs. S4-S6 is that generally $\chi_{\mathrm{c},GG}$ is similar to χ_{c,G_0G_0} in line shape with a smaller (by 20%) magnitude. χ_{c,G_0G_0} and $\chi_{\mathrm{c},GG}$ both show a dominant peak at $(0.5, 0.5)$, and a strongly suppressed double-peak structure along the $(Q_x, 0)$ direction. These line shapes are in huge contrast to χ_{c} from the numerically exact DCA

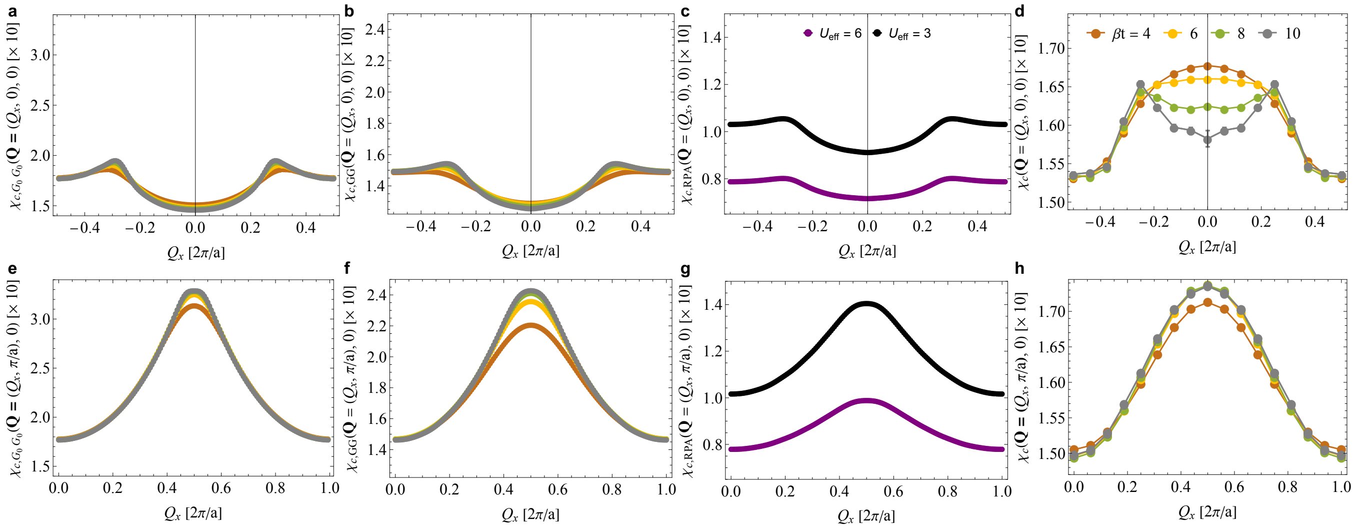

FIG. S4: The static charge susceptibility from the Lindhard function at $U/t = 0$ [panels **a**, **e**] and bubble approximation at $U/t = 6$ [panels **b**, **f**] with varying temperature. Panels **c** & **g** show the RPA result at the lowest temperature with two different U_{eff}. Panels **d** & **h** are a copy of Fig. 1**d** & **e** in the main text. The first row is for fixed $Q_y = 0$, and the second row is along $Q_y = 0.5$. All panels except **c** and **g** share the same legend. The filling and next-nearest-neighbor hopping are $\langle n \rangle = 1.2$ and $t' = -0.2t$ for all panels.

calculation. In the DCA results for $\chi_{\rm c}$ (far right panels d and h of Figs. S4-S6), the double peaks at fixed $Q_y = 0$ are comparable in magnitude to the $(0.5, 0.5)$ peak, which is significantly weaker than $\chi_{{\rm c},GG}$ (panels b and f of Figs. S4-S6). The RPA corrected results in panels c and g of Figs. S4-S6 are closer in magnitude to the DCA $\chi_{\rm c}$. However, the magnitude of the $(0.5, 0.5)$ peak is still much larger than that of the double peaks along $(Q_x, 0)$. Thus, none of the weak coupling approximations, $\chi_{{\rm c},G_0G_0}$, $\chi_{{\rm c},GG}$ and $\chi_{\rm c,RPA}$, captures the qualitative line shape of $\chi_{\rm c}$. They also fail to characterize the evolution from a single peak at high temperatures to the double peak structure (along $(Q_x, 0)$) at lower temperatures (Fig. S4). The same applies to the evolution with increasing carrier concentration (Fig. S5) or decreasing t' (Fig. S6).

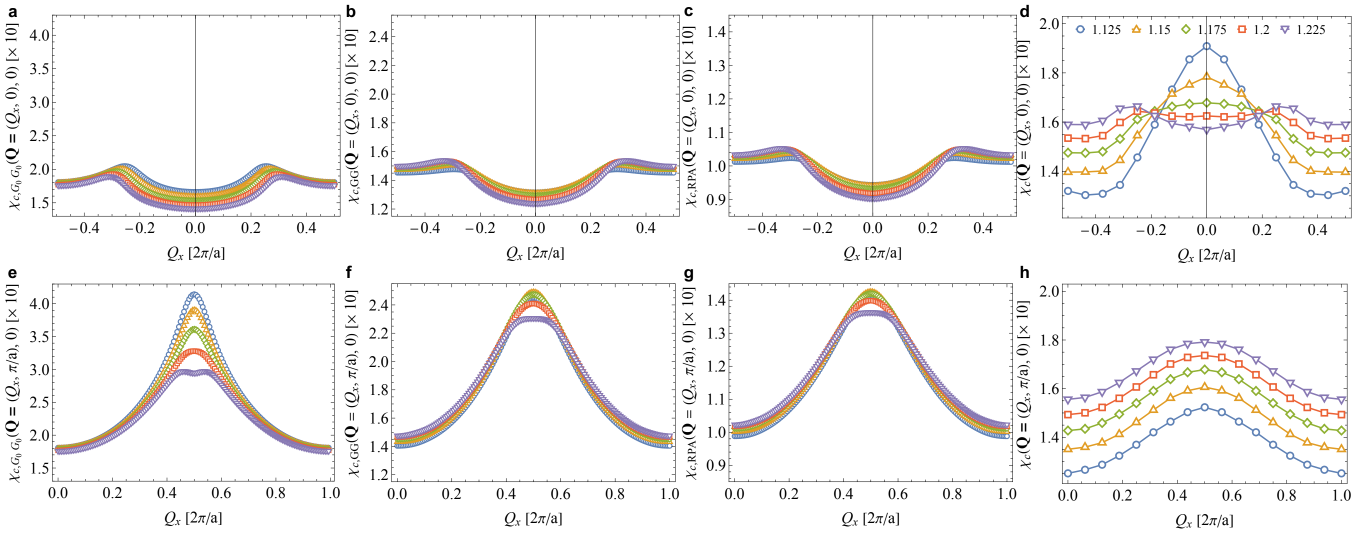

FIG. S5: The static charge susceptibility from the Lindhard function at $U = 0$ [panels **a**, **e**], bubble approximation at $U = 6$ [panels **b**, **f**] and RPA at $U_{\rm eff} = 3$ [panels **c**, **g**] with varying density. The first row is for fixed $Q_y = 0$, and the second row is along $Q_y = 0.5$ accordingly. Panels **d** is a copy of Fig. 3**a** in the main text while panel **h** shows the corresponding $\chi_c(\mathbf{Q}, 0)$ data long the (Q_x, π) direction. All panels share the same legend and are for fixed $\beta t = 8$, $t'/t = -0.2$.

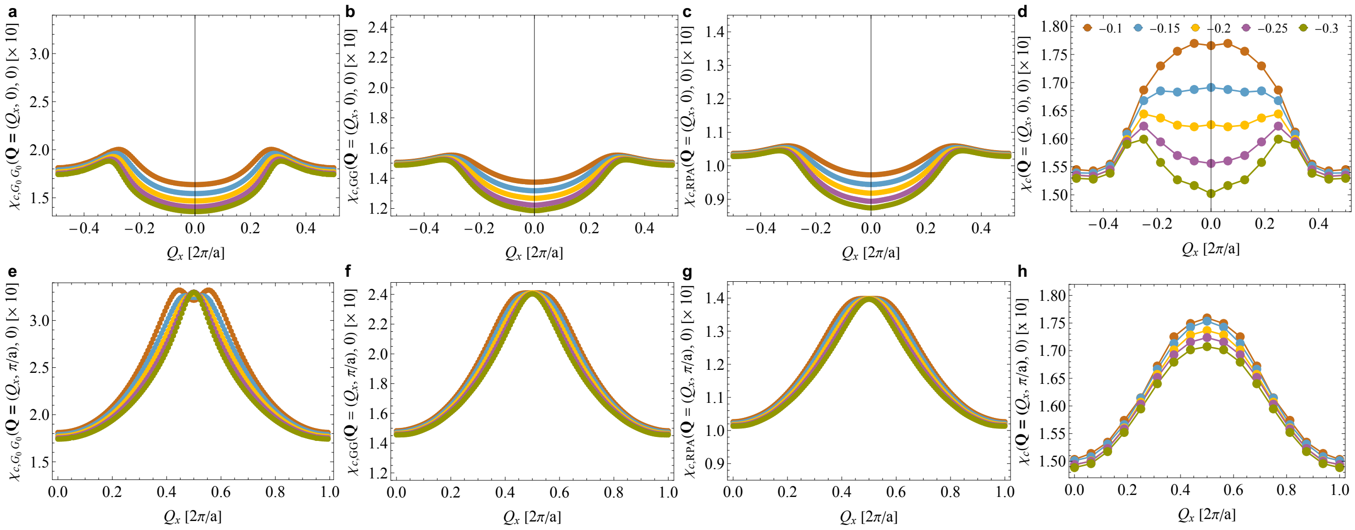

FIG. S6: The static charge susceptibility from the Lindhard function at $U = 0$ [panels **a**, **e**], bubble approximation at $U = 6$ [panels **b**, **f**] and RPA at $U_{\rm eff} = 3$ [panels **c**, **g**] with varying t'. The first row is for fixed $Q_y = 0$, and the second row is along $Q_y = 0.5$. Panel **d** is a copy of Fig. 3**a** in the main text while panel **h** shows the corresponding $\chi_c(\mathbf{Q}, 0)$ data long the (Q_x, π) direction. All panels share the same legend and are for fixed $\beta t = 8$, $\langle n \rangle = 1.2$.

In short, we find that both $\chi_{{\rm c},G_0G_0}$, $\chi_{{\rm c},GG}$ and $\chi_{\rm c,RPA}$ do not fully describe the qualitative features in the DCA results for $\chi_{\rm c}$. Specifically, these features include similar magnitude of the incommensurate peaks and the peak at

$(0.5, 0.5)$, the evolution of the double peak structure with decreasing temperature, as well as the appearance of a central peak with $Q_y = 0$ at smaller doping density and $|t'|$. This indicates that the physics of the CDW correlations in the e-doped Hubbard model is beyond that captured in weak coupling.

---

[1] N. S. Nichols, P. Sokol, and A. Del Maestro, Parameter-free differential evolution algorithm for the analytic continuation of imaginary time correlation functions, Phys. Rev. E **106**, 025312 (2022).

[2] M. Jarrell and J. Gubernatis, Bayesian inference and the analytic continuation of imaginary-time quantum Monte Carlo data, Physics Reports **269**, 133 (1996).

[3] F. Bao, Y. Tang, M. Summers, G. Zhang, C. Webster, V. Scarola, and T. A. Maier, Fast and efficient stochastic optimization for analytic continuation, Phys. Rev. B **94**, 125149 (2016).

[4] U. R. Hähner, T. A. Maier, and T. C. Schulthess, Continuous momentum dependence in the dynamical cluster approximation, Physical Review B **101**, 195114 (2020).

[5] T. A. Maier, M. Jarrell, and D. J. Scalapino, Spin susceptibility representation of the pairing interaction for the two-dimensional Hubbard model, Physical Review B **75**, 134519 (2007).

[6] T. A. Maier, A. Macridin, M. Jarrell, and D. J. Scalapino, Systematic analysis of a spin-susceptibility representation of the pairing interaction in the two-dimensional Hubbard model, Physical Review B **76**, 144516 (2007), publisher: American Physical Society.
